# TERT rs2736100 genotypes are associated with differential risk of myeloproliferative neoplasms in Swedish and Chinese male patient populations

**DOI:** 10.1007/s00277-016-2787-7

**Published:** 2016-08-25

**Authors:** Jenny Dahlström, Tiantian Liu, Xiaotian Yuan, Leonie Saft, Mehran Ghaderi, Ya Bin Wei, Catharina Lavebratt, Ping Li, Chengyun Zheng, Magnus Björkholm, Dawei Xu

**Affiliations:** 1 Department of Medicine, Division of Hematology and Center for Molecular Medicine (CMM), Karolinska Institutet, Karolinska University Hospital Solna, Stockholm, 17177 Sweden; 2 Department of Pathology, Shandong University School of Medicine, Jinan, China; 3 Department of Oncology and Pathology, Karolinska Institutet, Stockholm, Sweden; 4 Department of Molecular Medicine and Surgery, Neurogenetics Unit, Karolinska Institutet, Stockholm, Sweden; 5 Shandong University School of Nursing, Jinan, China; 6 Department of Hematology, The Second Hospital of Shandong University, Jinan, China

**Keywords:** Telomerase, Myeloproliferative neoplasms, Cancer genetics, Single nucleotide polymorphism, TERT

## Abstract

The *telomerase reverse transcriptase* (*TERT*) gene rs2736100_C allele has recently been shown to be associated with an increased risk for myeloproliferative neoplasms (MPNs) among Caucasians. However, it is unknown if this association is present in other ethnical populations and whether rs2736100 allele frequencies mirror the incidence of MPNs in a population. Here we genotyped TERT rs2736100 variants in 126 Swedish and 101 Chinese MPN patients and their age-, sex-, and ethnically-matched healthy controls. Healthy Chinese adults had a higher frequency of the A allele and lower frequencies of the C allele compared to Swedish counterparts (57.4 vs 47.0 % for A, 42.6 vs 53.0 % for C, *P* = 0.006). Both Swedish and Chinese patients harbored significantly higher C allele frequency than their controls (62.7 vs 53.0 % and 57.4 vs 42.6 % for Swedish and Chinese, respectively, *P* = 0.004). Swedes and Chinese bearing the CC genotype had a significantly increased risk of MPN compared to AA carriers (OR = 2.47; 95 % CI: 1.33–4.57, *P* = 0.003, for Swedes, and OR = 3.45; 95 % CI: 1.52–7.85, *P* = 0.005, for Chinese). Further analyses showed that rs2736100_CC was associated with robustly enhanced risk in males only (CC vs AA, OR = 5.11; 95 % CI: 2.19–11.92, *P* < 0.0001). The CC-carrying MPN patients exhibited significantly higher TERT expression than patients with the AC genotype. Collectively, the rs2736100_C is a risk allele for MPNs in Swedish and Chinese males, and the lower incidence of MPNs in the Chinese population is correlated with a lower rs2736100_C risk allele frequency.

## Introduction

Philadelphia chromosome negative myeloproliferative neoplasms (MPNs), consisting of polycythemia vera (PV), essential thrombocythemia (ET), and primary myelofibrosis (PMF), are characterized by hyperproliferation in the bone marrow leading to an excessive amount of mature cells from one or more of the myeloid lineages [1]. MPNs generally have a relatively indolent course, representing an early stage of leukemogenesis. However, a subset of patients eventually evolves into myelodysplastic syndrome (MDS) or acute myeloid leukemia (AML) [1–3]. Several genetic alterations have been identified in MPNs, with the most common being the JAK2^V617F^ mutation seen in 95 % of PV and 50 % of ET and PMF. Other genetic aberrations include calreticulin (CALR) and thrombopoetin receptor (MPL) mutations that are present in 70 and 10 % of JAK2^V617F^-negative ET and PMF, respectively [1, 4–6]. The identification of these mutations provides profound insights into the pathogenesis of MPNs, but the exact etiology remains incompletely understood. For instance, the MPN incidence varies significantly dependent on geographical areas, from approximately 2/100,000 person-years in China [7] to 5.8/100,000 person-years in Europe [1, 8], which highlights differences in genetic susceptibility. However, little is known about genetic events underlying this difference in incidence between Caucasians and Chinese.

Telomerase, a RNA-dependent DNA polymerase with telomerase reverse transcriptase (TERT) as its key catalytic component, is silent in most normal human cells due to the transcriptional repression of the *TERT* gene, whereas TERT induction coupled with telomerase activation is required for malignant transformation [9, 10]. The aberrant TERT expression confers malignant cells infinite proliferative potential. Similarly, high telomerase activity and TERT expression has been observed in MPNs [11, 12]. More recently, a number of studies uncovered an association between the rs2736100 A>C single nucleotide polymorphism (SNP) in the *TERT* gene and the risk of developing MPNs [13–17]. The rs2736100 is located at intron 2 of the *TERT* locus, and its CC genotype was previously observed to enhance TERT transcription, thereby increasing cancer risk [18]. However, so far, all the data of the rs2736100 association with MPN risk have been exclusively obtained from the analysis of Caucasians. It is currently unclear whether this is the case in the Chinese population or if there is a racial disparity. Moreover, given the significant difference in MPN incidence between Western and Chinese populations, it is important to determine whether the distribution of rs2736100 genotypes differs between these two ethnic groups. In the present study, we aimed to address these issues by genotyping rs2736100 in both Swedish and Chinese MPN patients and their corresponding healthy controls.

## Materials and methods

### Study populations

One hundred one Chinese MPN patients and 101 age- and sex-matched healthy adults were recruited from Shandong University Hospitals, China. Patients diagnosed with MPN (*N* = 126) at the Karolinska University Hospital in Solna, Stockholm, were also recruited to this study. The SNP rs2736100 has recently been studied in a Swedish general population [19], and an age- and sex-matched part of this population (*n* = 756) was used as control for the Swedish MPN patients. The study was approved by the Shandong University Second Hospital Ethics Committee and Stockholm Regional Ethics Review Board, and informed consent was obtained from all the participants.

### DNA extraction and SNP genotyping

DNA was extracted from blood derived from Swedish and Chinese MPN patients, and Chinese healthy controls using QIAmp DNA blood kit (Qiagen, Hilden, Germany). For Swedish healthy controls, saliva was collected and DNA extracted using a whole-saliva collection device (Oragene•DNA sample collection kit; DNA Genotek Inc., Canada) [19]. DNA concentration was measured on a NanoDrop 2000 spectrophotometer (Thermo Scientific, Waltham, MA, USA). Genotyping of rs2736100 was performed using pre-designed TaqMan SNP genotyping assay kits on a QuantStudio 7 flex system (Applied Biosystems, Waltham, MA, USA) as previously described [20]. The assay included negative controls and was run with the following condition: 95 °C for 10 min followed by 40 cycles of 92 °C for 15 s and 60 °C for 1 min. The genotyping success rate was >95 %.

### RNA isolation and quantitative reverse transcription-PCR (qRT-PCR)

Total cellular RNA in granulocytes from MPN patients was isolated using TRIzol reagent (Life Technologies, Carlsbad, CA, USA). Two micrograms of RNA was used for reverse transcription using M-MLV (Life Technologies, Carlsbad, CA, USA)) according to the manufacturer’s recommendation. Real-time amplification was performed in triplicate using SYBR Green PCR Master Mix (Life Technologies, Carlsbad, CA, USA) with QuantStudio 7 Flex Teal-Time PCR system (Applied Biosystems, Waltham, MA, USA), and the following primers were used: TERT, 5′-CGGAAGAGTGTCTGGAGCAA-3′ (forward) and 5′-GGATGAAGCGGAGTCTGGA-3′ (reverse); β2-M, 5′-GAATTGCTATGTGTCT GGGT-3′ (forward) and 5′-CATCTTCAAACCTCCATGATG-3′ (reverse). Levels of TERT messenger RNA (mRNA) were calculated from threshold cycle values and normalized to β2-M mRNA abundance, and expressed as arbitrary units.

### Telomere length analysis with flow-FISH

Telomere length in granulocytes from the Swedish MPN patients was measured using flow-FISH as previously described [21]. In short, fluorescent PNA probes (Panagene, Daejeon, Korea) were hybridized to the telomere sequence and the fluorescent signal was measured using a Gallios flow cytometer (Beckman Coulter, Brea, CA, USA) and analyzed using the Kaluza software (Beckman Coulter, Brea, CA, USA). Fluorescent MESF-FITC beads (Bangs Laboratories, Fishers, IN, USA) were used and the fluorescent signal was quantified using the QuickCal v.2.3 data analysis program (Bangs Laboratories, Fishers, IN, USA).

### Statistical analysis

Age and sex differences between patients and controls were compared using Mann-Whitney *U* and Fisher’s exact test, respectively. Differences in telomere length and TERT mRNA levels among different genotype groups were determined using Mann-Whitney *U* test. Fisher’s exact test was used to determine odds ratio with 95 % confidence interval (CI) and *P* value. All statistical analyses were performed in GraphPad Prism. *P* values <0.05 were considered statistically significant.

## Results

### Demographic and clinical characteristics of study subjects

A total of 126 MPN patients from Sweden and 101 from China were genotyped for rs2736100 variants. Clinical features including age, sex, MPN subtype, and mutation status (JAK2^V617F^ and CALR mutations) are summarized in Table 1. Seven hundred fifty-six Swedish and 101 Chinese healthy age- and sex-matched adults were used as controls (Table 1). ET were more common and PV more uncommon among the Chinese patients than the Swedish patients. The Chinese patients were also younger than the Swedish patients.

**Table 1:** Characteristics of healthy controls and patients with MPN

	Sweden		China	
	Controls	MPN	Controls	MPN
Number	756	126	101	101
Age (years)				
Mean ± SD	64 ± 5	64 ± 14	58 ± 15	58 ± 15
Median (range)	64 (54–74)	65 (25–106)	60 (17–82)	60 (17–82)
Sex [%females]	53	53	50	50
MPN subtype, *n* (%)				
PV		41 (32.5)		16 (15.8)
ET		40 (31.7)		38 (37.6)
PMF		28 (22.3)		15 (14.9)
MPN-NOS		17 (13.5)		32 (31.7)
JAK2-status,* n* (%)				
JAK2 V617F^−^		60 (47.6)		38 (37.6)
JAK2 V617F^+^		66 (52.4)		56 (55.4)
Unknown		0		7 (7.0)
CALR mutation		45		Unknown

*MPN* myeloproliferative neoplasm, *PV* polycythemia vera, *ET* essential thrombocythemia, *PMF* primary myelofibrosis

### Different rs2736100 allele distributions in healthy Swedish and Chinese populations

We first compared the distribution of rs2736100 genotypes in the healthy Swedish and Chinese control populations. As shown in Table 2, the Chinese population had lower C and higher A allele frequencies than the Swedish population (42.6 vs 53.0 % for C and 57.4 vs 47.0 % for A, respectively, *P* = 0.006). To verify whether this finding was representative, we further collected published genotyping data from another cohort of 289 healthy adults from the Shandong area (north) [22] and one from south of China (Shanghai and Guangzhou areas) [18]. Both of them showed a rs2736100 genotype distribution very similar to that of the present study (Table 3). The rs2736100 allele/genotype distribution in healthy populations from Sweden and other European countries (south and east) [15, 16] was also compared to the healthy populations in China. All of the previously published studies displayed largely similar rs2736100 variant frequency, with significantly higher C while lower A alleles in European countries (48.0 vs 57.4 % and 52.0 vs 42.6 % for A and C, respectively, *P* < 0.001). There was no difference in the AC genotype between Chinese and Caucasians (Tables 2 and 3).

**Table 2:** Comparison of genotype distribution of TERT rs2736100 in healthy control populations in Sweden and China

		Sweden	China	
***rs2736100***		N (%)	N (%)	*P value*
Genotype		756 (100)	101 (100)	
	AC+CC	589 (77.9)	68 (67.3)	*0.024*
	AA	167 (22.1)	33 (32.7)	
	AA+CC	379 (50.1)	51(50.5)	0.916
	AC	377 (49.9)	50 (49.5)	
	AA+AC	544 (72.0)	83 (82.2)	*0.031*
	CC	212 (28.0)	18 (17.8)	
Allele	A	711 (47.0)	116 (57.4)	*0.006*
	C	801 (53.0)	86 (42.6)	

OR and P-value generated using Fishers' exact test

Significant p-values are shown in italic

**Table 3:** Published rs2736100 genotype distributions of healthy populations in China and Europe

Author	Number	AA (%)	AC (%)	CC (%)	A (%)	C (%)	Area	Reference
China								
Dahlström et al.	101	33 (32.7)	50 (49.5)	18 (17.8)	116 (57.4)	86 (42.6)	North^a^	This study
Yuan et al.	289	86 (29.8)	144 (49.8)	59 (20.4)	316 (54.7)	262 (45.3)	North^a^	[18]
Wei et al.	2520	814 (32.3)	1269 (50.4)	437 (17.3)	2897 (57.5)	2143 (42.5)	South^b^	[16]
Total	2910	933 (32.1)*	1463 (50.3)*	514 (17.6)*	3329 (57.2)*	2491 (42.8)*		
Europe								
Dahlström et al.	756	167 (22.1)	377 (49.9)	212 (28.0)	711 (47.0)	801 (53.0)	Sweden	This study
Jäger et al.	202	47 (23.3)	88 (43.6)	67 (33.2)	182 (45.0)	222 (55.0)	Italy	[14]
Krahling et al.	400	111 (27.8)	188 (47.0)	101 (25.2)	410 (51.3)	390 (48.7)	Hungary	[13]
Total	1358	325 (23.9)**	653 (48.0)**	380 (28.1)**	1303 (48.0)**	1413 (52.0)**		
		AA vs AC + CC	AC vs AA + CC	CC vs AA + AC		C vs A		
*P* value (* vs **)		<0.001	0.106	<0.001		<0.001		

OR and *P* value were generated using chi-squared test

^a^From Shandong area

^b^From Shanghai and Guangzhou areas

### Rs2736100_C allele association with risk of MPN

Given different distributions of the rs2736100 allele frequency between control populations from two different countries, we chose to analyze the Swedish and Chinese MPN patients separately. The rs2736100_C allele frequency was significantly higher in both Swedish and Chinese MPN patients than in their corresponding controls (62.7 vs 53.0 % and 57.4 vs 42.6 % for Swedish and Chinese patients, respectively, *P* = 0.004) (Table 4). Compared to the AA variant carriers, Swedish and Chinese patients bearing the CC genotype exhibited significantly increased risk of MPN (OR = 2.47; 95 % CI: 1.33–4.57, *P* = 0.003, for Swedish, and OR = 3.45; 95 % CI: 1.52–7.85, *P* = 0.005, for Chinese patients) (Table 4). The AC genotype was also associated with an increased risk of MPN, but the *P* value for Chinese was at borderline significance, likely due to relatively few patients (AC vs AA: OR = 2.02; 95 % CI: 1.00–4.08, *P* = 0.057, for Chinese, and OR = 1.89; 95 % CI: 1.05–3.41, *P* = 0.034, for Swedish). Higher frequency of C alleles or CC genotypes was observed in all MPN subtypes, and there was no significant difference among patients with PV, ET, and PMF, respectively.

**Table 4:** Comparison of TERT rs2736100 genotypes in MPN patients and healthy controls

*rs2736100* genotype	Sweden				China			
	Control	MPN			Control	MPN		
	*n* (%)	*n* (%)	OR (95 % CI)	*P*	*n* (%)	*n* (%)	OR (95 % CI)	*P*
All	756 (100)	126 (100)			101 (100)	101 (100)		
Alleles								
A	711 (47.0)	94 (37.3)	1.0 (ref)		116 (57.4)	86 (42.6)	1.0 (ref)	
C	801 (53.0)	158 (62.7)	1.49 (1.13-1.96)	*0.004*	86 (42.6)	116 (57.4)	1.82 (1.23-2.70)	*0.004*
Genotypes								
AA	167 (22.1)	15 (11.9)	1.0 (ref)		33 (32.7)	17 (16.8)	1.0 (ref)	
AC	377 (49.9)	64 (50.8)	1.89 (1.05-3.41)	*0.034*	50 (49.5)	52 (51.5)	2.02 (1.00-4.08)	0.057
CC	212 (28.0)	47 (37.3)	2.47 (1.33–4.57)	*0.003*	18 (17.8)	32 (31.7)	3.45 (1.52–7.85)	*0.005*
AA + AC	544 (72.0)	79 (62.7)	1.0 (ref)		83 (82.2)	69 (68.3)	1.0 (ref)	
CC	212 (28.0)	47 (37.3)	1.53 (1.03–2.27)	*0.044*	18 (17.8)	32 (31.7)	2.14 (1.11–4.14)	*0.033*
AC + CC	589 (77.9)	111 (88.1)	1.0 (ref)		68 (67.3)	84 (83.2)	1.0 (ref)	
AA	167 (22.1)	15 (11.9)	0.48 (0.27–0.84)	*0.009*	33 (32.7)	17 (16.8)	0.42 (0.21–0.81)	*0.014*

OR and *P* value were generated using Fishers’ exact test

*MPN* myeloproliferative neoplasms, *OR* odds ratio, *CI* confidence interval

Significant *p*-values are shown in italic

### rs2736100_C allele association with risk of MPN in males

As shown in Table 5, there was no difference in the rs2736100 allele distribution between control males and females. However, MPN patients displayed a sex-dependent variant difference: male patients carried significantly more C alleles than control males did (66.8 vs 52.5 %, *P* < 0.001), and the AC and CC genotypes were associated with increased risk of MPNs in males compared to the AA variant (OR = 3.48; 95 % CI: 1.53–7.91, *P* = 0.002, for AC; OR = 5.11; 95 % CI: 2.19–11.92, *P* < 0.0001, for CC) (Table 5). In contrast, no differences in rs2736100 variants were observed between female controls and female patients (Table 5). Within the patient population, men were less likely to harbor the AA variant (OR = 0.25; 95 % CI: 0.10–0.61, *P* < 0.001) whereas no difference was seen in the healthy population (Table 5).

**Table 5:** Genotypes of TERT rs2736100 in men and women

*rs2736100 *genotype	Men				Women			
	Control		MPN		Control		MPN	
	*n* (%)	*n* (%)	OR (95 % CI)	*P*	*n* (%)	*n* (%)	OR (95 % CI)	*P*
All	405 (100)	110 (100)			452 (100)	117 (100)		
A	385 (47.5)	73 (33.2)	1.0 (ref)		442 (48.9)	107 (45.7)	1.0 (ref)	
	425 (52.5)	147 (66.8)	1.82 (1.33–2.49)	<0.001	462 (51.1)	127 (54.3)	1.14 (0.85–1.52)	0.420
AA	87 (21.5)	7 (6.4)	1.0 (ref)		113 (25.0)	25 (21.4)	1.0 (ref)	
AC	211 (52.1)	59 (53.6)	3.48 (1.53–7.91)	0.002	216 (47.8)	57 (48.7)	1.19 (0.71–2.01)	0.601
CC	107 (26.4)	44 (40.0)	5.11 (2.19–11.92)	<0.0001	123 (27.2)	35 (29.9)	1.29 (0.72–2.28)	0.469
	Control				MPN			
	Men		Women		Men		Women	
AC + CC	318 (78.5)	339 (74.3)	1.0 (ref)		103 (93.6)	92 (78.6)	1.0 (ref)	
AA	87 (21.5)	113 (25.7)	0.82 (0.60–1.13)	0.227	7 (6.4)	25 (21.4)	0.25 (0.10–0.61)	*0.001*

OR and *P* value were generated using Fisher’s exact test

*OR* odds ratio, *CI* confidence interval

### Rs2736100 variant association with JAK2^V617F^ and CALR mutations in MPNs

The relationship between major molecular subtypes in MPN (JAK2^V617F^ and CALR mutations) and rs2736100 variants was analyzed. The association between CALR and rs2736100 was only studied in the Swedish MPN patients. There was no association between any specific TERT rs2736100 variant and the presence of JAK2^V617F^. Patients harboring CALR mutations (insertion or deletion) (*n* = 45) tended to have a lower frequency of the CC variant compared to those carrying wt CALR, but the difference did not reach a significant level (OR = 0.44; 95 % CI: 0.20–1.01, *P* = 0.07).

### The relationship of rs2736100 variants with TERT expression and telomere length in MPNs

**Fig. 1:**
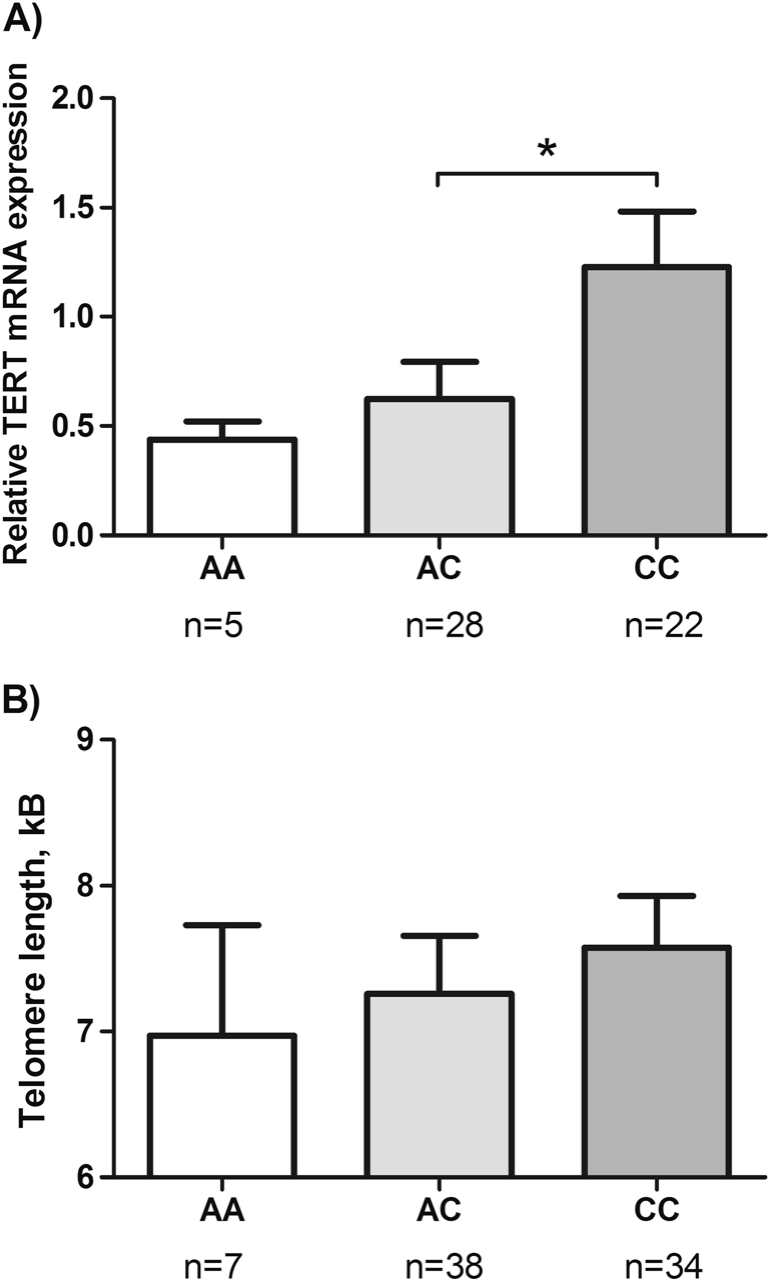
TERT mRNA expression and telomere length in MPN patients with different TERT rs2736100 variants. Total cellular RNA and genomic DNA were extracted from granulocytes derived from Swedish MPN patients harboring different TERT rs2736100 genetic variants. TERT mRNA and telomere length were measured by using qRT-PCR and flow-FISH, respectively. **a** TERT mRNA expression and (**b**) telomere length. *P < 0.05

TERT mRNA expression and leukocyte telomere length was previously assessed in the Swedish cohort of MPN patients [21], and we thus further determined a potential impact of different rs2736100 genotypes on TERT expression and telomere homeostasis. Patients bearing the CC genotype expressed the highest levels of TERT mRNA compared to those with AA and AC variants. There was a significant difference between AC- and CC-carrying patients (*P* = 0.024). The lowest TERT expression was observed in patients with AA genotype, but the difference was not significant, likely due to too few patients (*N* = 5). In MPN patients there was no correlation between rs2736100 allele variant and telomere length (Fig. 1).

## Discussion

The TERT rs2736100_C allele has recently been identified to be associated with increased risk of MPNs based on analyses of Caucasian patients [13–16]. This finding provides significant insights into genetic susceptibility to MPN. However, a number of issues remain to be defined: first, it is well known that racial disparities exists in both germline and somatic genetic alterations in the pathogenesis of cancer [22]. Thus, it is important to evaluate whether the correlation between rs2736100 allele variant and risk of MPN is also observed in other ethnic populations. Second, it is currently unclear whether the influence of TERT rs2736100_C allele on MPN susceptibility is identical in both males and females. Third, there is a threefold difference in MPN incidence between European countries and China, but the underlying mechanism is unclear. Is there a link between rs2736100 frequency distribution and MPN incidence? Finally, abnormal telomere length and structure is widespread in MPNs [11, 21], and it is unknown whether different rs2736100 variants influence TERT expression and telomere length differentially in MPNs. To answer these questions we recruited healthy adults and MPN patients from both Sweden and China for rs2736100 genotyping. The results presented here demonstrate that (i) the rs2736100_C allele is significantly associated with susceptibility to MPNs in both Swedish and Chinese individuals, but the association was restricted to male patients; (ii) the Chinese healthy population bears significantly lower rs2736100_C but higher A alleles than their Swedish counterpart, which is highly consistent with the lower incidence of MPNs in China; (iii) the rs2736100_CC-carrying Swedish patients display highest TERT expression in their myeloid cells.

The accumulated evidence suggests a close connection between germline genetic variation and susceptibility to human diseases. Given TERTs critical role in carcinogenesis, SNPs at the *TERT* locus have been extensively analyzed for their association with cancer risk, among which rs2736100 variants are most studied [18, 20, 23–25]. Indeed, the rs2736100_C allele is reported to increase the risk of multiple types of cancer [18, 20, 23–25]. It is believed that the CC genotype promotes TERT transcription, thereby upregulating telomerase activity and maintaining telomere length required for unlimited proliferation of cancer cells [18]. Similarly, high telomerase activity in the bone marrow of patients with MPN has been reported [12]. Meanwhile, telomere shortening is well established in MPN myeloid cells [11, 21, 26], likely due to the hyperproliferation of these cells in MPNs. In this study, we did observe the highest TERT expression in granulocytes derived from MPN patients bearing the CC genotype. These results indicate that the C allele may be able to upregulate TERT expression and in turn compensate for overerosion of telomere length in MPNs. In addition, TERT possesses many other important activities independent of its telomere-lengthening function [27–30], which has been implicated in cancer development and progression. It is conceivable that these TERT effects may similarly contribute to MPN pathogenesis.

Racial or ethnic disparities in disease incidence and pathogenesis have been well documented, and different genetic backgrounds are believed to play important parts. Likewise, there is a significant difference in the MPN incidence between Han Chinese (2/100,000) and European Caucasians (5.8/100,000) [8]. However, the underlying mechanism remains poorly understood. In the present study, we found that the rs2736100_C allele contributed to susceptibility to MPNs in both Swedish and Chinese populations. Moreover, the frequency of the A and C allele was significantly higher and lower, respectively, in Chinese controls than in Swedish and European Caucasian controls. These findings collectively reveal a positive correlation between MPN incidence and the rs2736100_C allele frequency. As rs2736100_C alleles are associated with an increased risk of many other malignancies [18, 20, 23–25], it is of interest to determine whether the same pattern can be identified in other malignancies where the incidence is lower in China than in Europe. In addition to TERT rs2736100, some other genetic variants have also been identified to be associated with MPN risk [17] and further comparison of their variant frequency between Chinese and Caucasians may contribute to a better understanding of germline variant effects on MPN incidence.

Intriguingly, our results showed a significant association of rs2736100_C with susceptibility to MPNs only in males. This observation is likely unbiased, because there is no difference in the rs2736100 genotype distribution between males and females in the general population in Sweden or China. Information on association between gender and allele variant of TERT rs2736100 in MPN patients is lacking in the majority of published studies [14–16], but one study reported a similar variant distribution in men and women [13]. Among MPN patients, women have been reported to have superior survival compared to men [31, 32]. One study reports that the shorter overall survival seen in male MPN patients is mainly due to increased frequency of secondary AML transformation [32], whereas another states that there are no gender differences in the risk of developing a secondary malignancy [33]. The CC genotype of TERT rs2736100 has also been associated with poorer response to anticancer agents and with cancer-promoting mutations, such as mutations in P53 [34]. Krahling et al. recently reported that MPN patients with the CC genotype had a higher probability to die from secondary solid tumors [15]. Taken together, these data indicate that the adverse outcome seen in male MPN patients may be associated with the rs2736100_CC genotype. Further studies are needed to verify and define the mechanistic role of rs2736100 variants in MPN progression and response to treatment.

We observed that the rs2736100_C allele was evenly distributed in all three subtypes of MPNs (PV, ET, and MF) with different genetic mutations (JAK2^V617F^ or CALR mutations). Similar findings were also documented in publications by Trifa et al. and others [13, 17]. These data collectively suggest that the C allele serves as a general risk factor for MPNs with no preferential susceptibility to a specific molecular subtype.

In summary, we demonstrate that the rs2736100_C allele is associated with increased risk to develop MPNs in both Swedish and Chinese populations. The direct comparison of the rs2736100 genotype between Swedish and Chinese healthy individuals shows a higher A but lower C allele frequency in Chinese individuals, which is correlated with a lower MPN prevalence in China. Further studies are required to dissect a causal relationship between the rs2736100_C allele and MPN development. These studies are needed to define how this allele contributes to MPN pathogenesis and to evaluate its possible role in AML transformation and disease progression.

## References

[1] Michiels JJ, Berneman Z, Schroyens W (2015). Changing concepts of diagnostic criteria of myeloproliferative disorders and the molecular etiology and classification of myeloproliferative neoplasms: from Dameshek 1950 to Vainchenker 2005 and beyond. Acta Haematol.

[2] Björkholm M, Derolf AR, Hultcrantz M (2011). Treatment-related risk factors for transformation to acute myeloid leukemia and myelodysplastic syndromes in myeloproliferative neoplasms. J Clin Oncol.

[3] Björkholm M, Hultcrantz M, Derolf Å (2014). Leukemic transformation in myeloproliferative neoplasms: therapy-related or unrelated?. Best Pract Res Clin Haematol.

[4] Nangalia J, Massie CE, Baxter EJ (2013). Somatic CALR mutations in myeloproliferative neoplasms with nonmutated JAK2. N Engl J Med.

[5] Langabeer SE, Andrikovics H, Asp J (2015). Molecular diagnostics of myeloproliferative neoplasms. Eur J Haematol.

[6] Klampfl T, Gisslinger H, Harutyunyan AS (2013). Somatic mutations of calreticulin in myeloproliferative neoplasms. N Engl J Med.

[7] http://haodf.health.sohu.com/disease/zhenxinghongxibaozengduozheng/jieshao.htm. 2009. Accessed 02 Aug 2016

[8] Hultcrantz M, Andersson TM-L, Landgren OM (2015). A population-based study of incidence of myeloproliferative neoplasms in Sweden between 2000 and 2012.

[9] Daniel M, Peek GW, Tollefsbol TO (2012). Regulation of the human catalytic subunit of telomerase (hTERT). Gene.

[10] Kong F, Zheng C, Xu D (2014). Telomerase as a “stemness” enzyme. Sci China Life Sci.

[11] Bernard L, Belisle C, Mollica L (2009). Telomere length is severely and similarly reduced in JAK2V617F-positive and -negative myeloproliferative neoplasms. Leukemia.

[12] Spanoudakis E, Bazdiara I, Pantelidou D (2011). Dynamics of telomere’s length and telomerase activity in Philadelphia chromosome negative myeloproliferative neoplasms. Leuk Res.

[13] Trifa AP, Bănescu C, Tevet M (2016). TERT rs2736100 A > C SNP and JAK2 46/1 haplotype significantly contribute to the occurrence of JAK2 V617F and CALR mutated myeloproliferative neoplasms—a multicentric study on 529 patients. Br J Haematol.

[14] Oddsson A, Kristinsson SY, Helgason H (2014). The germline sequence variant rs2736100_C in TERT associates with myeloproliferative neoplasms. Leukemia.

[15] Krahling T, Balassa K, Kiss KP (2016). Co-occurrence of Myeloproliferative Neoplasms and solid tumors is attributed to a Synergism between cytoreductive therapy and the common TERT Polymorphism rs2736100. Cancer Epidemiol Biomarkers Prev.

[16] Jäger R, Harutyunyan AS, Rumi E (2014). Common germline variation at the TERT locus contributes to familial clustering of myeloproliferative neoplasms. Am J Hematol.

[17] Tapper W, Jones AV, Kralovics R (2015). Genetic variation at MECOM, TERT, JAK2 and HBS1L-MYB predisposes to myeloproliferative neoplasms. Nat Commun.

[18] Wei R, Cao L, Pu H (2015). TERT Polymorphism rs2736100-C is associated with EGFR mutation-positive non-small cell lung cancer. Clin Cancer Res.

[19] Wei YB, Martinsson L, Liu JJ (2016). hTERT genetic variation in depression. J Affect Disord.

[20] Yuan X, Meng Y, Li P, et al (2016) The association between the TERT rs2736100 AC genotype and reduced risk of upper tract urothelial carcinomas in a Han Chinese population. Oncotarget 7:31972–31979 10.18632/oncotarget.7777 PMC5077989 26934125

[21] Dahlström J, Zhang X, Ghaderi M (2015). Dysregulation of shelterin factors coupled with telomere shortening in Philadelphia chromosome negative myeloproliferative neoplasms. Haematologica.

[22] Yuan X, Liu C, Wang K, et al (2016) The genetic difference between Western and Chinese urothelial cell carcinomas: infrequent FGFR3 mutation in Han Chinese patients. Oncotarget 7:25826–25835 10.18632/oncotarget.8404 PMC5041947 27029078

[23] Chen XF, Cai S, Chen QG (2012). Multiple variants of TERT and CLPTM1L constitute risk factors for lung adenocarcinoma. Genet Mol Res.

[24] Bojesen SE, Pooley KA, Johnatty SE (2013). Multiple independent variants at the TERT locus are associated with telomere length and risks of breast and ovarian cancer. Nat Genet.

[25] Mocellin S, Verdi D, Pooley KA (2012). Telomerase reverse transcriptase locus polymorphisms and cancer risk: a field synopsis and meta-analysis. J Natl Cancer Inst.

[26] Elena C, Rumi E, Portolan M (2011). Flow-FISH evaluation of telomere length in Philadelphia-negative myeloproliferative neoplasms. Haematologica.

[27] Ci X, Li B, Ma X (2015). Bortezomib-mediated down-regulation of telomerase and disruption of telomere homeostasis contributes to apoptosis of malignant cells. Oncotarget.

[28] Liu Z, Li Q, Li K (2013). Telomerase reverse transcriptase promotes epithelial-mesenchymal transition and stem cell-like traits in cancer cells. Oncogene.

[29] Ding D, Xi P, Zhou J (2013). Human telomerase reverse transcriptase regulates MMP expression independently of telomerase activity via NF-kB-dependent transcription. FASEB J.

[30] Singhapol C, Pal D, Czapiewski R (2013). Mitochondrial telomerase protects cancer cells from nuclear DNA damage and apoptosis. PLoS One.

[31] Hultcrantz M, Kristinsson SY, Andersson TM (2012). Patterns of survival among patients with myeloproliferative neoplasms diagnosed in Sweden from 1973 to 2008: a population-based study. J Clin Oncol.

[32] Stein B, Spivak JL, Moliterno AR (2015). Gender is a core modifier of disease outcomes and survival in the MPN.

[33] Ravn Landtblom A, Bower H, Andersson TM-L (2016). Increased risk of second malignancies in patients with myeloproliferative neoplasms diagnosed in Sweden 1973–2009—a population-based cohort study of 9,379 patients.

[34] Kim J, Jones-Hall YL, Wei R (2013). Association between hTERT rs2736100 polymorphism and sensitivity to anti-cancer agents. Front Genet.

